# Some Are More Equal - A Comparative Study on Swab Uptake and Release of Bacterial Suspensions

**DOI:** 10.1371/journal.pone.0102215

**Published:** 2014-07-10

**Authors:** Philipp Warnke, Liesa Warning, Andreas Podbielski

**Affiliations:** Institute of Medical Microbiology, Virology, and Hygiene, Rostock University Hospital, Rostock, Germany; Amphia Ziekenhuis, Netherlands

## Abstract

**Objectives:**

Swabs are widely used to collect samples for microbiological analyses from various clinical settings. They vary by material, size, and structure of the tip. This study investigates the uptake and release capacities for liquid and bacteria.

**Methods:**

Five swabs were analyzed for their uptake and release capacities of S*taphylococcus aureus* and *Staphylococcus epidermidis* suspensions. Two approaches were investigated providing volume-restricted and unrestricted amounts of bacterial suspensions to mimic various clinical situations. Volume and bacterial uptake and release were measured in milligrams and by counting colony forming units (CFU), respectively.

**Results:**

Volume uptake and release in the unrestricted setting varied highly significant between 239.6 mg and 88.7 mg (p<0.001) and between 65.2 mg and 2.2 mg (p<0.001), respectively. In the volume-restricted setting the complete volume was absorbed by all swabs, volume release could only be detected for flocked swabs (2.7 mg; p<0.001). Highest amount of CFU release was detected for the MWE Dryswab in the unrestricted setting for both *S. aureus* and *S. epidermidis* with 1544 CFU and 553 CFU, respectively, lowest release for the Sarstedt neutral swab with 32 CFU and 17 CFU, respectively (p<0.001). In the volume-restricted setting MWE Σ-Swab released the highest bacterial amount with 135 CFU *S. aureus* and 55 CFU *S. epidermidis*, respectively, the lowest amount was released by Mast Mastaswab with 2 CFU *S. aureus* and 1 CFU *S. epidermidis*, respectively (p<0.001). Within the range of the utilized bacterial concentrations, uptake/release ratios were identical for the particular swab types and independent of the bacterial species.

**Conclusions:**

The influence of the swab type on subsequent diagnostic results is often underestimated. Uptake and release of the investigated bacteria vary significantly between different swab types and sampling conditions. For best diagnostic outcome swabs should be chosen according to the examined situation and the swab performance profile.

## Introduction

Microbiological diagnostics are divided in three steps, i.e. (i) pre-analysis, (ii) analysis, and (iii) post-analysis. Optimum performance of the later steps is based on the performance of the previous ones. While steps (ii) and (iii) are exclusively in the hands of the laboratory, step (i) is typically performed at the patient and therefore, is at best partly under quality control of the laboratory. Thus, if the preanalytical step is performed with suboptimal quality, even the highest standards of laboratory quality management cannot compensate the initial flaws.

Swabs are frequently used to collect specimen for cultural as well as molecular microbiological analysis. Different swabs and transport systems have been evaluated for some in vitro and in vivo performance data in the past [1], [2], [3], [4], [5], [6], [7], [8]. Yet, in medical facilities often only one swab type is used to cover all needs for microbiological analysis, for example in screening tests for a bacterial carrier status, for the diagnosis of skin or mucosa infections, or to obtain specimens during operations. These situations completely differ in their general presence and amounts of body fluids. The upper limit for volume uptake of the swab is determined by its size and material. Common swab materials are for example rayon, Dacron, cotton, or nylon fibers or alternatively, cellular foam. The modes by which this material is applied to the swab shaft differs. While tips with wound fibers were used for decades, a novel swab type was recently introduced. On such swabs with so-called flocked tips the fibers are arranged in a perpendicular fashion with respect to its shaft, resulting in enhanced uptake and facilitated release of cellular specimens [9], [10], [11], [12].

Obviously, different tip materials have different physical and chemical characteristics which in turn individually influence the process of specimen collection. Additionally, swab handling techniques could significantly affect the recovery rates for microorganisms [13]. Thus, the seemingly simple process of sample collection by swab techniques is influenced by a complex array of parameters which, depending on additive or subtractive effects, could largely change the sensitivity of microbiological assays.

Aim of this study was to characterize swab type-dependent uptake and release rates for fluids and bacteria as important parameters of the preanalytical process in microbiological diagnostics. To address this issue, five commonly used swab types were tested in settings providing unrestricted and restricted volumes of bacterial, i.e. *S. aureus* and *S. epidermidis*, suspensions to mimic various clinical situations. Handling of the swabs followed a strict standardized protocol. Parameters analyzed were volume uptake and release as measured by weight in milligrams as well as uptake and release of different bacterial species as measured by CFU counting.

## Materials and Methods

### Swabs

The following swabs were tested:

1. MWE medical wire, Corsham Wiltshire England, Tubed Sterile Dryswab, rayon, ref. MW102;
2. MWE medical wire, Corsham Wiltshire England, Sigma Dry Swab Tubed, Σ-Swab, cellular foam, ref. MW941;
3. Mast Group Ltd., Reinfeld, Germany, MASTASWAB MD 555, via Copan, Brescia, Italy, ref. 800155;
4. Copan, Brescia, Italy, FLOQSwabs, nylon fiber, cat. no. 502CS01;
5. Sarstedt, Nuembrecht, Germany, neutral swab, rayon, via Copan, Brescia, Italy, cat.no. 80.1301.

### Bacterial culture techniques

*Staphylococcus aureus* strain ATCC 25923 and *Staphylococcus epidermidis* strain DSMZ 1798 were separately propagated at 37°C in Caso broth (Roth, Karlsruhe, Germany) as overnight standing cultures in ambient air. Early stationary phase cells were harvested, washed in phosphate-buffered saline (PBS; NaCl (137 mmol/l), KCl (2.7 mmol/l), Na_2_HPO_4_×2 H_2_O (10 mmol/l), KH_2_PO_4_ (2.0 mmol/l)) at pH 7.4 and resuspended in PBS+10% glycerol. Aliquots were stored at −80°C for up to 3 months. After 3 d conservation in the freezer, the viable cell count of 3 tubes was determined according to standard techniques. Bacterial suspensions were prepared for each test series at the day of usage, with a mixed suspension of *S. aureus* ATCC 25923 and *S. epidermidis* DSMZ 1798 bacterial strains at amounts of 2.6×10^4^ and 1×10^4^ colony-forming units (CFU)/ml, respectively.

### Experimental setting

#### Volume uptake and release

5 ml Falcon round-bottom tubes (Becton Dickinson, Heidelberg, Germany) were filled with 1000 µl or 10 µl bacteria suspended in PBS, respectively. To analyze volume uptake, swabs were placed into bacterial suspension for 15 seconds and then removed. After establishing the direct 1∶1 correlation of bacterial suspension volumes and masses by ten repeated measurements with a 0.1% error range (not shown), tube weight was quantified on a scale (Sartorius Excellence, Sartorius, Göttingen, Germany) prior to introduction and after removal of the swabs. To analyze volume release, inoculated swabs were placed into empty sterile round-bottom tubes, agitated at maximum speed on a lab mixer (Vortex Mixer, neoLab, Heidelberg, Germany) for 10 sec while exerting gentle pressure to the tube wall (swab shaft started to bend) and afterwards removed. Released volumes as measured by masses were then calculated by comparing tube weights before and after the procedure.

#### Bacterial uptake and release

In the unrestricted volume (1000 µl) setting, CFU were counted from the initial suspensions, the remaining volume and the released volume by plating 100 µl aliquots from ten-fold serial dilutions using PBS as the diluent on Columbia agar supplemented with 5% sheep blood (Becton Dickinson).

In the restricted volume (10 µl) setting, CFU were counted from the initial suspensions. As most of the swabs did not release any liquid, CFU were determined by streaking swabs in a standardized fashion onto Columbia agar supplemented with 5% sheep blood in 5 streaks with a length of 5 cm while constantly rotating the swab shaft in an angle of 45° to the plate and exerting gentle pressure (swab shaft was slightly bended).

For the elution of Copan FLOQSwabs according to manufacturer’s instructions, the swabs were rotated (10 turns) in eSwab liquid Amies preservation medium, Copan, Brescia, Italy, ref. 490CE.A. CFU were then counted by ten-fold serial dilution steps and plating of 200 µl aliquots onto Columbia agar supplemented with 5% sheep blood.

#### Iteration of experiments

All experiments were performed in triplicate (technical replicates) and repeated on three independent time points (biological replicates).

### Detection of bacteria

CFU of each bacterial strain were analyzed for every swab used in the experiments. Therefore, bacteria were cultured on Columbia agar supplemented with 5% sheep blood at 37°C under ambient atmosphere for 48 h. CFU were then counted by macroscopic inspection. *Staphylococcus aureus* was distinguished from *Staphylococcus epidermidis* by hemolysis (β-hemolysis vs. no hemolysis) and colony color (golden yellow vs. white), if necessary by agglutination assay (Slidex Staph Plus, bioMérieux, Marcy l’Etoile, France). Additionally, purity controls were performed by analyzing ten arbitrarily chosen colonies of five randomly selected plates with MALDI-TOF mass spectrometry (AXIMA Assurance, Shimadzu).

### Statistical analysis

Data were analyzed using nonparametric Mann-Whitney U test. All p values resulted from two-tailed statistical test. p-values of <0.05, <0.01, and <0.001 were considered to be marginally significant, significant, and highly significant, respectively.

Regarding reproducibility of the technical and biological replicates, we tested by using Kruskal-Wallis-Test for each parameter and received no significant results (significance, if p<0.01 because of Bonferroni correction for 5 tests, that means p<0.05/5).

### Ethics statement

The study was performed without using human or animal subjects and/or tissues.

For swabbing experiments of healthy humans (table S11) all volunteers provided written informed consent. This study was approved by the ethics committee of Rostock University Hospital (A 2014-0096).

## Results

To mimic different clinical situations, settings were chosen providing an unrestricted (1000 µl) and a restricted (10 µl) supply of bacterial suspensions. Since bacterial suspension volumes correlated to respective masses, volume uptake and release was measured in milligrams. Further, uptake and release of bacteria (for both settings) was analyzed by CFU counting.

### Volume-unrestricted setting (1000 µl)

#### Volume uptake

**Figure 1:**
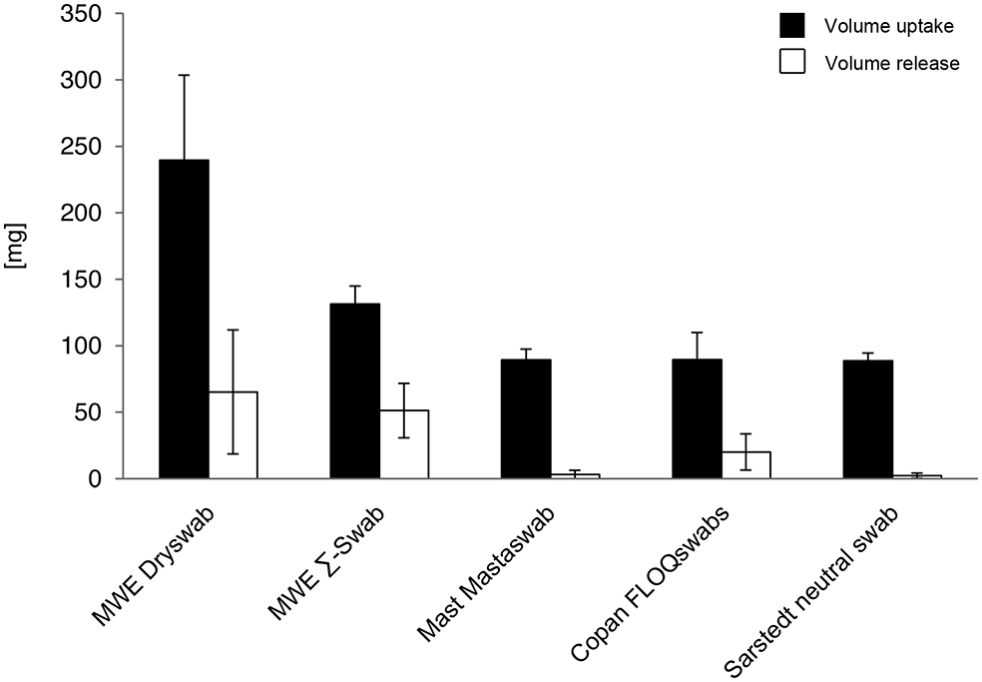
Volume uptake and release (volume-unrestricted setting). Volumes were determined by measuring weight [mg] of round-bottom tubes before and after uptake or deposition of fluids by 5 different swab types. Results from statistical analysis are shown in table S1.

Mean volume uptake by the different swab-types as measured by masses varied highly significant between 239.6 mg and 88.7 mg (p<0.001). Mean volume uptake by each swab-type was measured as followed: MWE Dryswab 239.6 mg; MWE Σ-Swab 131.3 mg; Mast Mastaswab 89.4 mg; Copan FLOQSwabs 89.7 mg; Sarstedt neutral swab 88.7 mg (figure 1, table S1 and table S9).

The highest amount was absorbed by the MWE Dryswab. The difference was highly significant compared to all other swabs (p<0.001).

The second highest amount was absorbed by the MWE Σ-Swab. Compared to Mast Mastaswab, Copan FLOQSwabs, and Sarstedt neutral swab the difference was highly significant (p<0.001). No significant difference in volume uptake could be observed between Mast Mastaswab, Copan FLOQSwabs, and Sarstedt neutral swab (p>0.05) (table S1).

#### Volume release

Mean volume release by the different swab-types varied highly significant between 65.2 mg and 2.2 mg (p<0.001). Mean volume release by each swab-type was measured as followed: MWE Dryswab 65.2 mg; MWE Σ-Swab 51.2 mg; Mast Mastaswab 3.1 mg; Copan FLOQSwabs 20.1 mg; Sarstedt neutral swab 2.2 mg (figure 1, table S1 and table S9).

The highest amount was released by the MWE Dryswab. The difference was highly significant compared to Mast Mastaswab and Sarstedt neutral swab (p<0.001), and significant compared to Copan FLOQSwabs (p<0.01). No significant difference in volume release could be observed compared to MWE Σ-Swab (p>0.05).

The second highest amount was released by the MWE Σ-Swab. Compared to Mast Mastaswab and Sarstedt neutral swab the difference was highly significant (p<0.001), and significant compared to Copan FLOQSwabs (p<0.01). Mast Mastaswab released highly significant less volume compared to Copan FLOQSwabs (p<0.001), and a similar amount compared to Sarstedt neutral swab (p>0.05). Differences between Copan FLOQSwabs and Sarstedt neutral swab were also highly significant (p<0.001) (table S1).

#### Release of bacteria in absolute numbers

**Figure 2:**
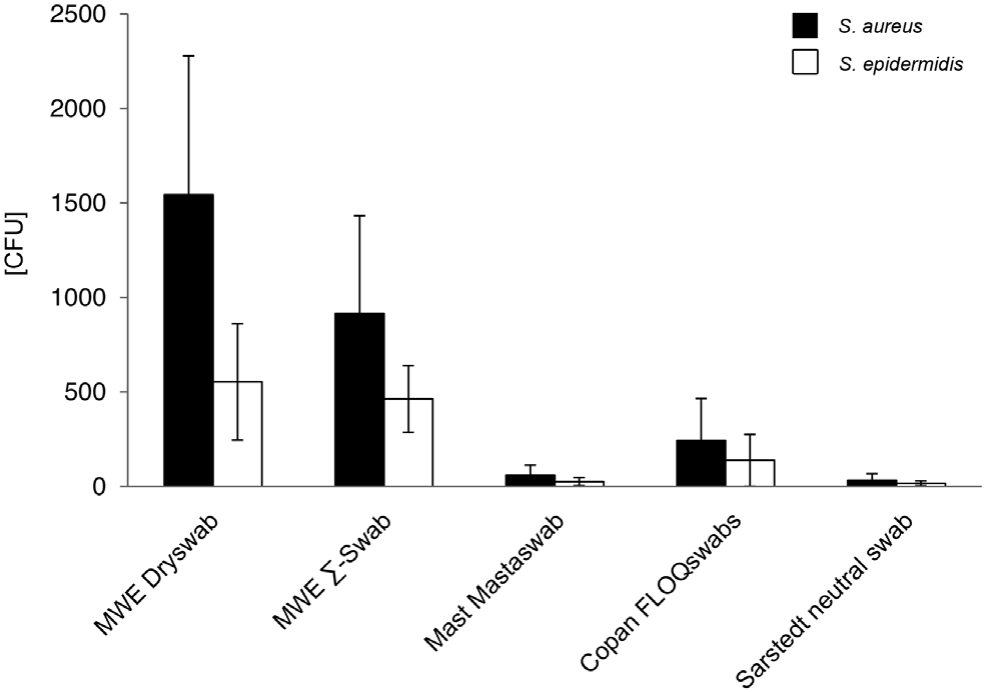
Release of bacteria in absolute numbers (volume-unrestricted setting). Viable counts of bacterial suspension after release by 5 different swab types were determined by serial dilutions and plate counting as described in the methods section. CFU = colony forming units. Results from statistical analysis are shown in table S2.

In this assay, each swab was immersed in a suspension containing 2.6±0.8×10^4^ CFU *S. aureus* and 1.0±0.2×10^4^ CFU *S. epidermidis* per ml. Mean release of bacteria by the different swab-types varied highly significant between 1544 to 32 CFU for *S. aureus* (p<0.001), and 553 to 17 CFU for *S. epidermidis*, respectively (figure 2, table S2 and table S9).

The following mean quantitative release for each swab-type was measured for *S. aureus*: MWE Dryswab 1544 CFU; MWE Σ-Swab 916 CFU; Mast Mastaswab 59 CFU; Copan FLOQSwabs 242 CFU; Sarstedt neutral swab 32 CFU (figure 2, table S9), and for *S. epidermidis*: MWE Dryswab 553 CFU; MWE Σ-Swab 463 CFU; Mast Mastaswab 25 CFU; Copan FLOQSwabs 138 CFU; Sarstedt neutral swab 17 CFU (figure 2, table S9). The highest bacterial numbers were released by the MWE Dryswab for both *S. aureus* and *S. epidermidis*. The differences were highly significant compared to Mast Mastaswab and Sarstedt neutral swab for both bacterial species (p<0.001), highly significant compared to Copan FLOQSwabs for *S. aureus* (p<0.001) and significant for *S. epidermidis* (p<0.01). Compared to MWE Σ-Swab the difference was only marginal significant for *S. aureus* (p<0.05) and not significant for *S. epidermidis* (p>0.05) (table S2).

The second highest bacterial amounts were released by the MWE Σ-Swab. Compared to Mast Mastaswab, Copan FLOQSwabs, and Sarstedt neutral swab the differences were highly significant for both bacterial species (p<0.001). Mast Mastaswab released significantly less bacteria compared to Copan FLOQSwabs (p<0.01), and a similar amount compared to Sarstedt neutral swab (p>0.05). Differences between Copan FLOQSwabs and Sarstedt neutral swab were also highly significant for *S. aureus* and *S. epidermidis*, respectively (p<0.001) (table S2).

#### Relative release of bacteria compared to uptake

**Figure 3:**
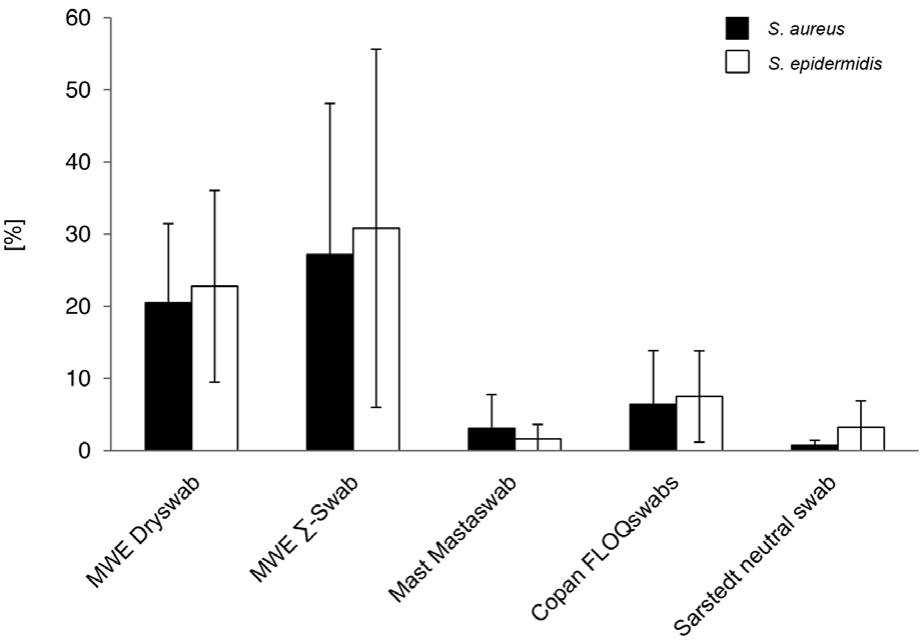
Relative bacterial release compared to initial uptake (volume-unrestricted setting). Ratios were determined by comparison of viable counts of absorbed and released bacteria. Results from statistical analysis are shown in table S3.

Mean release of bacteria compared to initial mean uptake varied swabtype-dependent between 27.2% and 0.7% for *S. aureus* and 30.8% and 1.6% for *S. epidermidis*, respectively (figure 3). Since statistical analysis was performed on absolute CFU counts (figure 2, table S2), it was not repeated on the relative ratios.

With respect to the two staphylococcal species, the relative uptake/release ratios did not show significant differences, thereby confirming nonselective interaction of the tip material with the bacteria. Since *S. aureus* and *S. epidermidis* cells were used in a ratio of approximately 2.5: 1, within this range, the tip material did not display a dose-dependency for bacterial uptake or release (p>0.05) (table S3).

### Volume-restricted setting (10 µl)

#### Volume uptake

**Figure 4:**
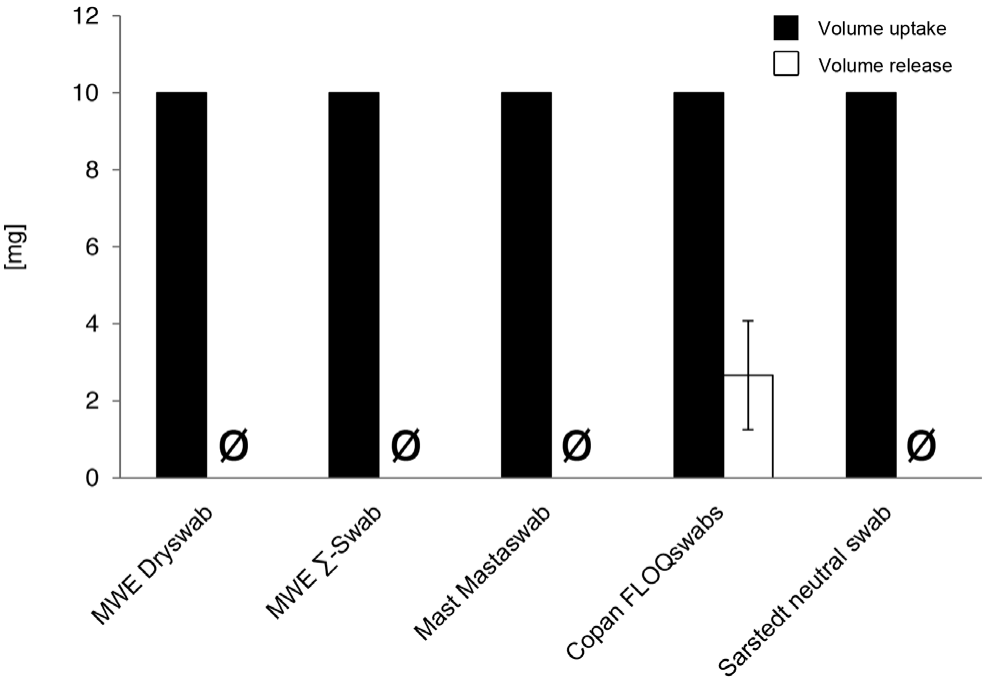
Volume uptake and release (volume-restricted setting). Volumes were determined by measuring weight [mg] of round-bottom tubes before and after uptake or deposition of fluids by 5 different swab types. Results from statistical analysis are shown in table S4.

All swabs completely absorbed the offered volume, thus no difference between the swabs could be observed (figure 4, table S4 and table S10).

#### Volume release

No volume was released by the swabs except for Copan FLOQSwabs, which released a mean mass of 2.7 mg. This was highly significant compared to the other swabs (p<0.001) (figure 4, table S4 and table S10).

#### Release of bacteria in absolute numbers

**Figure 5:**
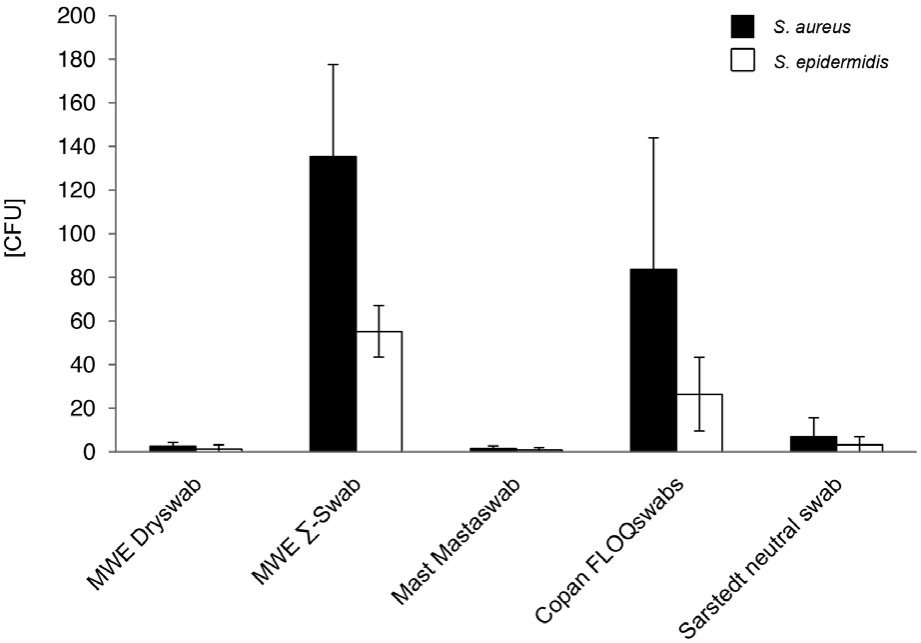
Release of bacteria in absolute numbers (volume-restricted setting). Viable counts of bacterial suspension after release by 5 different swab types were determined by serial dilutions and plate counting as described in the methods section. CFU = colony forming units. Results from statistical analysis are shown in table S5.

Since the complete offered volumes of bacterial suspensions were soaked up by the swaps, quantitation of absorbed bacteria was not performed, assuming that the major portion entered the tip material. Even when no volume could be extracted from most swab types, bacteria were expected to be released under typical diagnostic conditions. After directly streaking the swabs onto Columbia agar by a standardized technique, determined mean release of bacterial counts by the different swab-types ranged with highly significant differences for *S. aureus* and *S. epidermidis* from 135 to 2 CFU, and from 55 to 1 CFU, respectively (p<0.001) (figure 5, table S5 and table S10).

Mean counts for released *S. aureus* and *S. epidermidis* cells by each swab-type were: MWE Dryswab 3 CFU; MWE Σ-Swab 135 CFU; Mast Mastaswab 2 CFU; Copan FLOQSwabs 84 CFU; Sarstedt neutral swab 7 CFU and MWE Dryswab 1 CFU; MWE Σ-Swab 55 CFU; Mast Mastaswab 1 CFU; Copan FLOQSwabs 26 CFU; Sarstedt neutral swab 3 CFU, respectively (figure 5, table S10).

The highest bacterial numbers were released by the MWE Σ-Swab for both *S. aureus* and *S. epidermidis*. The differences were highly significant compared to MWE Dryswab, Mast Mastaswab, and Sarstedt neutral swab for both bacterial species (p<0.001), significant compared to Copan FLOQSwabs for *S. epidermidis* (p<0.01) and not significant for *S. aureus* (p>0.05) (table S5).

The second highest bacterial amounts were released by the Copan FLOQSwabs. Compared to MWE Dryswab, Mast Mastaswab, and Sarstedt neutral swab the bacterial release was significantly higher for both species (p<0.01), compared to the *S. aureus* release of Mast Mastaswab even highly significant (p<0.001). Between MWE Dryswab, Mast Mastaswab, and Sarstedt neutral swab no significant difference in the release of the two bacterial species could be observed (p>0.05) (table S5).

#### Relative release of bacteria compared to uptake

**Figure 6:**
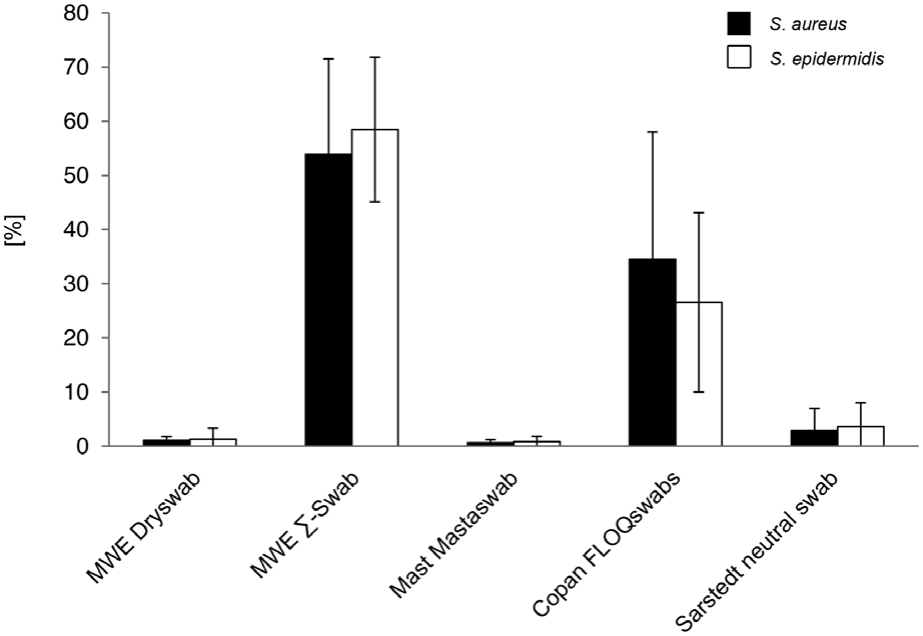
Relative bacterial release compared to initial uptake (volume-restricted setting). Ratios were determined by comparison of viable counts of absorbed and released bacteria. Results from statistical analysis are shown in table S6.

Mean release of bacteria compared to postulated initial uptake varied swabtype-dependent between 53.9% and 0.6% for *S. aureus* and 58.5% and 0.9% for *S. epidermidis*, respectively (figure 6). Again, statistical analysis was not performed here because already introduced for the corresponding absolute CFU numbers (figure 5, table S5).

Also again, relative release rates were species-independent for each swab-type. Considering a quantitative ratio of approximately 2.5: 1 between absorbed *S. aureus* and *S. epidermidis* cells, within this range a dose-dependent release could also be excluded (p>0.05) (table S6).

#### Release of bacteria into Amies preservation medium

**Figure 7:**
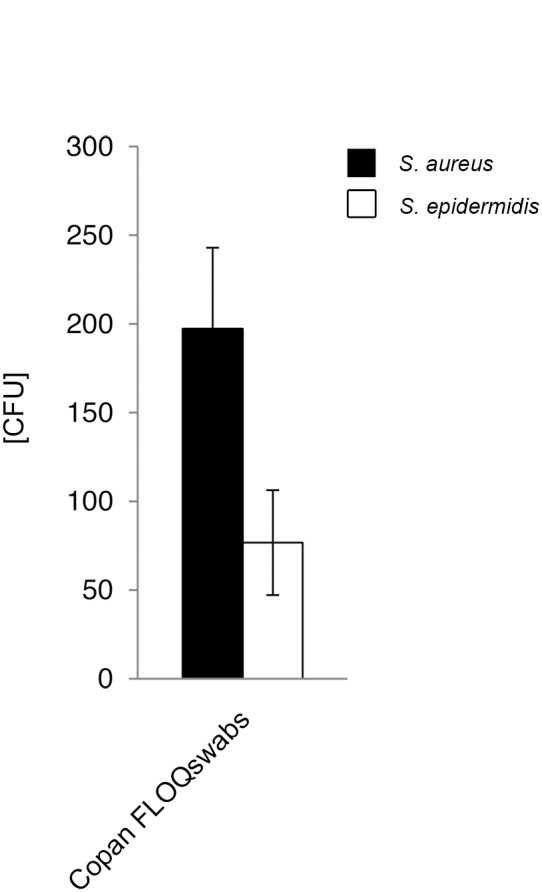
Bacterial release into Amies medium (volume-restricted setting). Viable counts of bacterial suspension after release by flocked swabs were determined by serial dilutions and plate counting as described in the methods section. CFU = colony forming units. Results from statistical analysis are shown in table S7.

**Figure 8:**
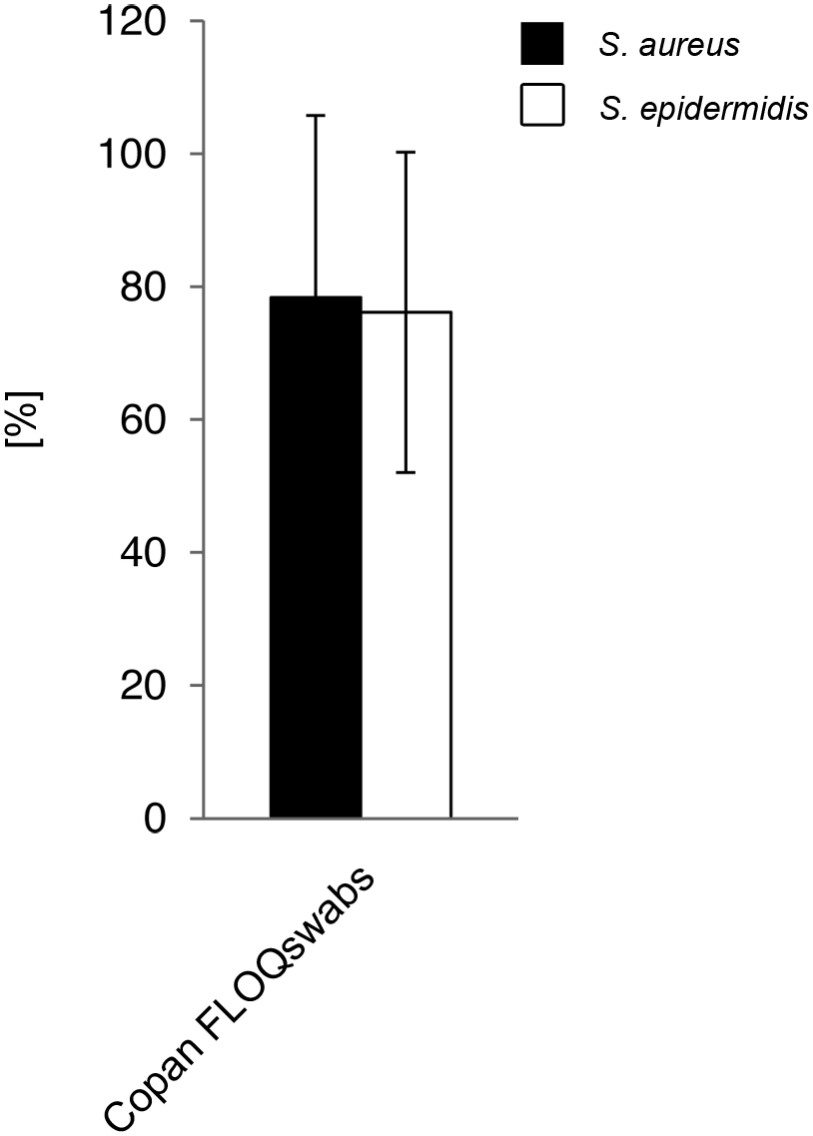
Relative bacterial release into Amies medium compared to initial uptake (volume-restricted setting). Ratios were determined by comparison of viable counts of absorbed and released bacteria. Results from statistical analysis are shown in table S8.

Release of bacterial numbers by rotating the swabs into Amies preservation medium was analyzed for Copan FLOQSwabs (figure 7, table S10) and compared to the released numbers using the same swab-type without medium. Mean release of 197 CFU *S. aureus* and 77 CFU *S. epidermidis* cells was significant higher, compared to 84 CFU and 26 CFU without medium, respectively (p<0.01) (table S7). Similar to the results obtained without using Amies medium, release rates were species- and dose-independent (p>0.05) (figure 8, table S8).

## Discussion

In contrast to massive recent changes in microbiological analysis and postanalysis due to the introduction of exciting novel techniques with high impact on test duration, sensitivity and specificity, the initial step of sample collection has undergone few if any principal changes. However, sample collection quality is crucial for the quality of the subsequent analytical steps and therefore, any improvements of this first step will benefit the whole diagnostic process. Although often leading to inferior diagnostic quality, sample collection utilizing swabs is the preferred technique for most clinicians because of its performance ease and swiftness. Thus, if the general approach for sample collection cannot be changed, it appears prudent to optimize swabs and swabbing techniques [13] especially with respect to test sensitivity. As a prerequisite for optimization, test sensitivities of presently available swabs should be quantified under conditions close to natural circumstances.

Swabs based on a new production technique, so called flocked swabs, have recently been introduced on a commercial basis. Several publications have shown the high performance rates of these swabs with respect to specimen uptake and release by in vitro and in vivo approaches [10], [14], [15], [16], [17], [18]. In the present study we intended to determine the test sensitivity of routinely used swabs produced by established procedures (i.e. fibers wound onto the swab tip), swabs with a foam tip and the flocked swabs by using two different protocols mimicking sample collection from comparatively dry surfaces such as skin and other epithelia (see table S11) and from wet surfaces such as operation wounds or operative sites [19], [20], conditions which so far have not been addressed in a single study.

In this study we separately monitored volume as well as bacterial uptake and release, since each parameter could differ between swabs due to unspecific interaction of fluids or microorganisms with the swab material. For test organisms, we chose *Staphylococcus aureus* as the most frequent causative agent of purulent infections on the skin or mucosa as well as in organ tissues and therefore diagnostic techniques should include detection of this species [21], [22], [23]. In addition, this species transiently or persistently colonizes especially the anterior nares of 20 to 30% of the human population and therefore is the target for the majority of screening assays for human carrier detection, especially in the nosocomial setting with respect to the MRSA-subset of *S. aureus* strains [21], [24], [25], [26], [27], [28], [29], [30], [31]. Since *S. aureus* is often associated with the resident microflora of skin and mucosa, we also included *S. epidermidis* [32], [33], [34], [35], [36], [37], [38] as the major representative species into our test format to monitor potential interactions affecting test sensitivity. The inoculum concentrations were between 10^3^–10^5^ CFU as recommended for swab evaluation testing by the Clinical and Laboratory Standards Institute for aerobic and facultative anaerobic bacteria [39]. To put emphasis on the clinical relevance of *S. aureus,* this strain was used in a ratio of 2.5: 1 compared to *S. epidermidis*, i.e. 2.6×10^4^ CFU/ml *S. aureus* and 1×10^4^ CFU/ml *S. epidermidis*, respectively, were offered in each test to the swabs.

Utilizing the unrestricted volume test format, absorbed and released volumes significantly differed between the tested swab types. The relative rates of absorbed and released bacterial numbers correlated quite well for each swab type, indicating that swab materials could behave differently towards fluid volumes and bacteria, and these differences comprise both the absorption and release steps. The differences between the swab types could add up to two orders of magnitude. Under these conditions, MWE Dryswab absorbed and released the highest amount of bacteria, while Mast Mastaswab and Sarstedt neutral swab carried the lowest amounts. This indicated, that in a clinical setting with ample fluid supply, swabs with the highest liquid uptake rates such as MWE Dryswab and MWE Σ-Swab would perform best with respect to highest diagnostic yield.

These conclusions cannot be applied to clinical settings with restricted volume amounts. In such a setting, only the Copan FLOQSwabs released some fluid. When testing bacterial release upon contact to an agar surface, only MWE Σ-Swabs and Copan FLOQSwabs released mentionable bacterial numbers. Thus, under such conditions and in the presence of only low bacterial counts, usage of MWE Dryswab, Mast mastaswab and Sarstedt neutral swab could lead to false negative results. Under routine diagnostic conditions, most microbiological specimens are processed by semiquantitative methods [40]. For instance, in a recent study, nasal colonization was categorized as low, 1+, 2+, 3+, and 4+. Then low colonization was described in 31% of patients [41], a situation when the above mentioned disclaimer for the latter three swab types would apply.

The performance of Copan FLOQSwabs under volume-restricted conditions was further improved by applying the tip into Amies medium directly after the absorption of bacterial suspensions as also described when employing other bacterial species [42]. With this specific protocol, this swab type led to the best results – similar to the results of an in vivo study on swabs for nasal MRSA screening [43]. However, this protocol requires usage of the complete set of swab and transport tubes offered by the manufacturer. If the tubes are not available or the laboratory procedures are not set to deal with liquid specimens, MWE Σ-Swabs offer a similarly efficient alternative. The present experimental settings did not stress the swab surface during absorption of bacterial suspensions. Contact to rough surfaces obviously affects the tip structures to a varying degree (more so for wound fibers than for flocked ones), which in turn would additionally effect bacterial recovery rates in a swab type-dependent manner [44]. That phenomenon would additionally favor usage of flocked swabs for examination of (relatively) dry surfaces. However, the advantage of flocked swaps for test sensitivity is limited to such surfaces and does not extend to situations of ample surface supply.

Importantly, for all tested swab types bacterial release was staphylococcal species and, within the tested range, dose independent. Thus, frequent *S. epidermidis* presence in skin, mucosa and wound material should not affect detection of the most important pathogen *S. aureus*.

There are limitations to this study. Here we focused on bacterial species important in MRSA screening examinations. Yet, for some of tested swab types the results apparently apply to other bacterial species [42], although that study used bacterial suspensions in volumes between the extreme boundaries of the present study. Also, bacterial release was analyzed shortly after inoculation of the tips to simulate optimum transport conditions, thereby omitting potential interactions between tip material and drying bacteria during variable transport periods [45]. Suboptimum transport conditions lead to reduced survival rates of bacteria and consecutively to a decline in analytical sensitivity [2], [46], [47]. This would especially affect swab types with low numbers of collected bacteria. However, examination of these circumstances would dramatically increase the complexity of results because of the different transport vials and media used in the clinical setting.

## Conclusion

This study emphasizes the role of preanalytical parameters on the corresponding diagnostic results. Commonly used swabs vary significantly with respect to uptake and release of liquid and bacteria. Definitely, the clinical setting influences the results obtained by swabbing specimens and should be considered when choosing a swab-type. Obviously, one swab type does not fit all needs and careful selection of swab types for the specific clinical setting plus thorough education of clinical staff for choice of the appropriate swab type as well as its proper usage appears to be necessary for optimal diagnostic results in clinical microbiology.

## Supporting Information

Table S1 Volume uptake and release (volume-unrestricted setting). All p values result from nonparametric, two-tailed Wilcoxon-Mann-Whitney U-test.
(DOCX)

Table S2 Release of bacteria in absolute numbers (volume-unrestricted setting). All p values result from nonparametric, two-tailed Wilcoxon-Mann-Whitney U-test. CFU = colony forming units.
(DOCX)

Table S3 Relative bacterial release compared to initial uptake (volume-unrestricted setting). All p values result from nonparametric, two-tailed Wilcoxon-Mann-Whitney U-test.
(DOCX)

Table S4 Volume uptake and release (volume-restricted setting). All p values result from nonparametric, two-tailed Wilcoxon-Mann-Whitney U-test.
(DOCX)

Table S5 Release of bacteria in absolute numbers (volume-restricted setting). All p values result from nonparametric, two-tailed Wilcoxon-Mann-Whitney U-test. CFU = colony forming units.
(DOCX)

Table S6 Relative bacterial release compared to initial uptake (volume-restricted setting). All p values result from nonparametric, two-tailed Wilcoxon-Mann-Whitney U-test.
(DOCX)

Table S7 Bacterial release into Amies medium compared to direct plating (volume-restricted setting). All p values result from nonparametric, two-tailed Wilcoxon-Mann-Whitney U-test. CFU = colony forming units.
(DOCX)

Table S8 Relative bacterial release into Amies medium compared to initial uptake (volume-restricted setting). All p values result from nonparametric, two-tailed Wilcoxon-Mann-Whitney U-test.
(DOCX)

Table S9 Mean values of volume and bacterial uptake and release (volume-unrestricted setting). CFU = colony forming units.
(DOCX)

Table S10 Mean values of volume and bacterial uptake and release (volume-restricted setting). CFU = colony forming units. n.d. = not detected.
(DOCX)

Table S11 Amounts of specimen uptaken by pharynx-, nose-, and skin-swabs. 3 healthy volunteers were swabbed at different anatomical locations as indicated. Specimen uptake was measured in milligrams [mg]. SD = standard deviation.
(DOCX)
