# Supplementary material for: Some Are More Equal - A Comparative Study on Swab Uptake and Release of Bacterial Suspensions
Source: PLoS One. 2014 Jul 10;9(7):e102215. doi: 10.1371/journal.pone.0102215 (PMC4092111; doi:10.1371/journal.pone.0102215)
Supplement: Table S2 — Release of bacteria in absolute numbers (volume-unrestricted setting). All p values result from nonparametric, two-tailed Wilcoxon-Mann-Whitney U-test. CFU = colony forming units. (DOCX) [file pone.0102215.s002.docx]

**Table S2. Release of bacteria in absolute numbers (volume-unrestricted setting).**

Legend: All p values result from nonparametric, two-tailed Wilcoxon-Mann-Whitney U-test. CFU = colony forming units.

|  | CFU release  *S. aureus* | CFU release  *S. epidermidis* |
| --- | --- | --- |
| MWE Dryswab vs. MWE Σ-Swab | p<0.05 | p=0.67 |
| MWE Dryswab vs. Mast Mastaswab | p<0.001 | p<0.001 |
| MWE Dryswab vs. Copan FLOQswabs | p<0.001 | p<0.01 |
| MWE Dryswab vs. Sarstedt neutral swab | p<0.001 | p<0.001 |
| MWE Σ-Swab vs. Mast Mastaswab | p<0.001 | p<0.001 |
| MWE Σ-Swab vs. Copan FLOQswabs | p<0.001 | p<0.001 |
| MWE Σ-Swab vs. Sarstedt neutral swab | p<0.001 | p<0.001 |
| Mast Mastaswab vs. Copan FLOQswabs | p<0.01 | p<0.01 |
| Mast Mastaswab vs. Sarstedt neutral swab | p=0.22 | p=0.60 |
| Copan FLOQswabs vs. Sarstedt neutral swab | p<0.001 | p<0.001 |
