# Supplementary material for: Some Are More Equal - A Comparative Study on Swab Uptake and Release of Bacterial Suspensions
Source: PLoS One. 2014 Jul 10;9(7):e102215. doi: 10.1371/journal.pone.0102215 (PMC4092111; doi:10.1371/journal.pone.0102215)
Supplement: Table S3 — Relative bacterial release compared to initial uptake (volume-unrestricted setting). All p values result from nonparametric, two-tailed Wilcoxon-Mann-Whitney U-test. (DOCX) [file pone.0102215.s003.docx]

**Table S3. Relative bacterial release compared to initial uptake (volume-unrestricted setting).**

Legend: All p values result from nonparametric, two-tailed Wilcoxon-Mann-Whitney U-test.

|  | *S. aureus* vs.  *S. epidermidis* |
| --- | --- |
| MWE Dryswab | p=0.80 |
| MWE Σ-Swab | p=0.67 |
| Mast Mastaswab | p=0.81 |
| Copan FLOQswabs | p=0.49 |
| Sarstedt neutral swab | p=0.14 |
