# Supplementary material for: Some Are More Equal - A Comparative Study on Swab Uptake and Release of Bacterial Suspensions
Source: PLoS One. 2014 Jul 10;9(7):e102215. doi: 10.1371/journal.pone.0102215 (PMC4092111; doi:10.1371/journal.pone.0102215)
Supplement: Table S4 — Volume uptake and release (volume-restricted setting). All p values result from nonparametric, two-tailed Wilcoxon-Mann-Whitney U-test. (DOCX) [file pone.0102215.s004.docx]

**Table S4. Volume uptake and release (volume-restricted setting).**

Legend: All p values result from nonparametric, two-tailed Wilcoxon-Mann-Whitney U-test.

|  | Volume uptake | Volume release |
| --- | --- | --- |
| MWE Dryswab vs. MWE Σ-Swab | p=1.00 | p=1.00 |
| MWE Dryswab vs. Mast Mastaswab | p=1.00 | p=1.00 |
| MWE Dryswab vs. Copan FLOQswabs | p=1.00 | p<0.001 |
| MWE Dryswab vs. Sarstedt neutral swab | p=1.00 | p=1.00 |
| MWE Σ-Swab vs. Mast Mastaswab | p=1.00 | p=1.00 |
| MWE Σ-Swab vs. Copan FLOQswabs | p=1.00 | p<0.001 |
| MWE Σ-Swab vs. Sarstedt neutral swab | p=1.00 | p=1.00 |
| Mast Mastaswab vs. Copan FLOQswabs | p=1.00 | p<0.001 |
| Mast Mastaswab vs. Sarstedt neutral swab | p=1.00 | p=1.00 |
| Copan FLOQswabs vs. Sarstedt neutral swab | p=1.00 | p<0.001 |
