# Supplementary material for: Some Are More Equal - A Comparative Study on Swab Uptake and Release of Bacterial Suspensions
Source: PLoS One. 2014 Jul 10;9(7):e102215. doi: 10.1371/journal.pone.0102215 (PMC4092111; doi:10.1371/journal.pone.0102215)
Supplement: Table S6 — Relative bacterial release compared to initial uptake (volume-restricted setting). All p values result from nonparametric, two-tailed Wilcoxon-Mann-Whitney U-test. (DOCX) [file pone.0102215.s006.docx]

**Table S6. Relative bacterial release compared to initial uptake (volume-restricted setting).**

Legend: All p values result from nonparametric, two-tailed Wilcoxon-Mann-Whitney U-test.

|  | *S. aureus* vs.  *S. epidermidis* |
| --- | --- |
| MWE Dryswab | p=0.55 |
| MWE Σ-Swab | p=0.44 |
| Mast Mastaswab | p=0.73 |
| Copan FLOQswabs | p=0.55 |
| Sarstedt neutral swab | p=0.93 |
