# Supplementary material for: Some Are More Equal - A Comparative Study on Swab Uptake and Release of Bacterial Suspensions
Source: PLoS One. 2014 Jul 10;9(7):e102215. doi: 10.1371/journal.pone.0102215 (PMC4092111; doi:10.1371/journal.pone.0102215)
Supplement: Table S7 — Bacterial release into Amies medium compared to direct plating (volume-restricted setting). All p values result from nonparametric, two-tailed Wilcoxon-Mann-Whitney U-test. CFU = colony forming units. (DOCX) [file pone.0102215.s007.docx]

**Table S7. Bacterial release into Amies medium compared to direct plating (volume-restricted setting).**

Legend: All p values result from nonparametric, two-tailed Wilcoxon-Mann-Whitney U-test. CFU = colony forming units.

|  | CFU release  *S. aureus* | CFU release  *S. epidermidis* |
| --- | --- | --- |
| Amies medium vs. direct plating | p<0.01 | p<0.01 |
