# Supplementary material for: Some Are More Equal - A Comparative Study on Swab Uptake and Release of Bacterial Suspensions
Source: PLoS One. 2014 Jul 10;9(7):e102215. doi: 10.1371/journal.pone.0102215 (PMC4092111; doi:10.1371/journal.pone.0102215)
Supplement: Table S9 — Mean values of volume and bacterial uptake and release (volume-unrestricted setting). CFU = colony forming units. (DOCX) [file pone.0102215.s009.docx]

**Table S9. Mean values of volume and bacterial uptake and release (volume-unrestricted setting).**

Legend: CFU = colony forming units.

|  | Volume uptake  [mg] | Volume release  [mg] | CFU uptake  *S. aureus* | CFU release  *S. aureus* | CFU uptake  *S. epidermidis* | CFU release  *S. epidermidis* |
| --- | --- | --- | --- | --- | --- | --- |
| MWE Dryswab | 239.6 ± 64.0 | 65.2 ± 46.6 | 8868 ± 4784 | 1544 ± 735 | 3294 ± 2066 | 553 ± 308 |
| MWE Σ-Swab | 131.3 ± 13.5 | 51.2 ± 20.5 | 6633 ± 5839 | 916 ± 517 | 3130 ± 2799 | 463 ± 177 |
| Mast Mastaswab | 89.4 ± 8.0 | 3.1 ± 3.3 | 7701 ± 7676 | 59 ± 54 | 3001 ± 2273 | 25 ± 21 |
| Copan FLOQSwabs | 89.7 ± 20.3 | 20.1 ± 13.7 | 5975 ± 3758 | 242 ± 224 | 2395 ± 1690 | 138 ± 137 |
| Sarstedt neutral swab | 88.7 ± 5.8 | 2.2 ± 1.9 | 7113 ± 5454 | 32 ± 34 | 2487 ± 2765 | 17 ± 12 |
