# Supplementary material for: Some Are More Equal - A Comparative Study on Swab Uptake and Release of Bacterial Suspensions
Source: PLoS One. 2014 Jul 10;9(7):e102215. doi: 10.1371/journal.pone.0102215 (PMC4092111; doi:10.1371/journal.pone.0102215)
Supplement: Table S10 — Mean values of volume and bacterial uptake and release (volume-restricted setting). CFU = colony forming units. n.d. = not detected. (DOCX) [file pone.0102215.s010.docx]

**Table S10. Mean values of volume and bacterial uptake and release (volume-restricted setting).**

Legend: CFU = colony forming units. n.d. = not detected.

|  | Volume uptake  [mg] | Volume release  [mg] | CFU uptake  *S. aureus* | CFU release  *S. aureus* | CFU uptake  *S. epidermidis* | CFU release  *S. epidermidis* | CFU release  *S. aureus*  (Amies medium) | CFU release  *S. epidermidis*  (Amies medium) |
| --- | --- | --- | --- | --- | --- | --- | --- | --- |
| MWE Dryswab | 10.0 | 0.0 | 262 ± 85 | 3 ± 2 | 97 ± 20 | 1 ± 2 | n.d. | n.d. |
| MWE Σ-Swab | 10.0 | 0.0 | 262 ± 85 | 135 ± 42 | 97 ± 20 | 55 ± 12 | n.d. | n.d. |
| Mast Mastaswab | 10.0 | 0.0 | 262 ± 85 | 2 ± 1 | 97 ± 20 | 1 ± 1 | n.d. | n.d. |
| Copan FLOQSwabs | 10.0 | 2.7 ± 1.4 | 262 ± 85 | 84 ± 60 | 97 ± 20 | 26 ± 17 | 197 ± 46 | 77 ± 30 |
| Sarstedt neutral swab | 10.0 | 0.0 | 262 ± 85 | 7 ± 9 | 97 ± 20 | 3 ± 4 | n.d. | n.d. |
