# Supplementary material for: Some Are More Equal - A Comparative Study on Swab Uptake and Release of Bacterial Suspensions
Source: PLoS One. 2014 Jul 10;9(7):e102215. doi: 10.1371/journal.pone.0102215 (PMC4092111; doi:10.1371/journal.pone.0102215)
Supplement: Table S11 — Amounts of specimen uptaken by pharynx-, nose-, and skin-swabs. 3 healthy volunteers were swabbed at different anatomical locations as indicated. Specimen uptake was measured in milligrams [mg]. SD = standard deviation. (DOCX) [file pone.0102215.s011.docx]

**Table S11. Amounts of specimen uptaken by pharynx-, nose-, and skin-swabs.**

Legend: 3 healthy volunteers were swabbed at different anatomical locations as indicated. Specimen uptake was measured in milligrams [mg]. SD = standard deviation.

|  |  |  | Specimen uptake [mg] |  |
| --- | --- | --- | --- | --- |
|  | **Volunteer** | **Pharynx** | **Nose** | **Skin** |
| MWE Dryswab | 1 | 24 | 5 | 1 |
|  | 2 | 23 | 13 | 0 |
|  | 3 | 19 | 11 | 1 |
| MWE Σ-Swab | 1 | 15 | 4 | 2 |
|  | 2 | 26 | 6 | 1 |
|  | 3 | 20 | 9 | 0 |
| Mast Mastaswab | 1 | 15 | 13 | 0 |
|  | 2 | 13 | 20 | 2 |
|  | 3 | 20 | 11 | 1 |
| Copan FLOQswabs | 1 | 27 | 13 | 1 |
|  | 2 | 23 | 9 | 0 |
|  | 3 | 18 | 8 | 1 |
| Sarstedt neutral swab | 1 | 16 | 12 | 0 |
|  | 2 | 28 | 7 | 1 |
|  | 3 | 40 | 15 | 1 |
| Mean ± SD |  | **21.8 ± 6.8** | **10.4 ± 4.2** | **0.8 ± 0.7** |
| Total mean ± SD  (pharynx, nose, skin) |  |  | **11.0 ± 9.8** |  |
